# Data of *de novo* assembly of the leaf transcriptome in *Aegle marmelos*

**DOI:** 10.1016/j.dib.2018.05.095

**Published:** 2018-05-23

**Authors:** Prashant Kaushik, Shashi Kumar

**Affiliations:** a Instituto de Conservación y Mejora de la Agrodiversidad Valenciana, Universitat Politècnica de València, Valencia, Spain; b International Center for Genetic Engineering and Biotechnology, Aruna Asaf Ali Marg, New Delhi 110067, India

**Keywords:** *De novo* assembly, Transcriptome, Indian Bael, *Aegle marmelos*

## Abstract

*Aegle marmelos* (Indian Bael) is an important plant from the religious and medicinal point of view. Many medicinal compounds are identified from its leaves that rendered its use in traditional as well as modern medical system. Still, it is quite overlooked from the transcriptome viewpoint. This article provides information about the transcriptomic data which is the first ever report about this plant. The data is accessible via NCBI BioProject (id PRJNA433585).

**Specifications Table**

**Table:** 

Subject area	*Plant Biology*
More specific subject area	*Transcriptomics*
Type of data	*Assembly of reads and sequence annotation*
How data was acquired	*cDNA sequencing was performed using Illumina HiSeq. 2500*
Data format	*Raw (FASTQ) sequences*
Experimental factors	*N.A.*
Experimental features	*Leaves of Aegle marmelos cultivar “Kaghzi” were used for RNA extraction then to generate paired end libraries with HiSeq. 2500 system. This data generated was further utilized for doing transcriptome assembly using Trinity*
Data source location	*Kurukshetra, India*
Data accessibility	*Accessible as NCBI BioProject (PRJNA433585).*https://www.ncbi.nlm.nih.gov/bioproject/?term=PRJNA433585

**Value of the data**

• *Aegle marmelos* is a member of genus Aegle this data set is the first report about this plant.
• This data will be helpful to perform phylogenetic analysis and to perform a new analysis by using different approaches.
• This data is also applicable to identify different pathways in the *Aegle marmelos* leaves as it is also an important medicinal plant and leaves are well exploited for their use to treat various ailments.

## 1 Data

*Aegle marmelos*, known as Indian Bael or Golden Apple, is a member of Rutaceae or citrus family. The plants are mostly cultivated on marginal lands due to their drought tolerance. *Aegle marmelos* is a mid-sized tree around 10 to 15-meter height and bears small-medium sized edible fruits. The leaves are trifoliate pale green in colour. Besides its importance as a medicine in tradition, the leaves are reported to possess anti-diabetic, anti-inflammatory, anti-microbial, anti-lipidemic and hepatoprotective activities which are attributed to the presence of several compounds identified in the leaves such as aegeline, marmelosin, skimmianine, and umbelliferone [1], [2], [3], [4], [5]. Here the information about the leaf transcriptomic data generated using Illumina HiSeq. 2500 is provided.

## 2 Experimental design, materials, and methods

Young leaves from three healthy and approximately 5-year-old plants of *Aegle marmelos* variety “Kaghzi” were collected in August of 2017 from the Government Garden Nursery Kurukshetra, India. The sampled leaves tissues were trimmed to around 5 mm in dimension and were stored in the RNAlater (Life Technologies, USA). The RNA of all the three individuals was extracted separately using standard TRIzol reagent (Invitrogen, USA) around 150 mg of leaf sample was crushed to the powdered form in liquid nitrogen by mortar and pestle that was further agitated with 1 ml of TRIzol reagent [6], [7]. The quality and quantity check of the extracted RNA was carried out by Agilent 2100 Bioanalyzer and Nanodrop ND-1000 spectrophotometer (Nanodrop Technologies, Montchain, DE, USA) respectively. The RNA was pooled from all the three individuals in equimolar concentration to make one representative sample for sequencing. TruSeq RNA Library Prep Kit v2 from Illumina® (Illumina, Inc., USA) was used for library preparation and library quantification was performed using Qubit Fluorometer (Qubit™ dsDNA HS Assay Kit). The cDNA library was sequenced on Illumina HiSeq. 2500 (2×125 bp) platform.

**Fig. 1:**
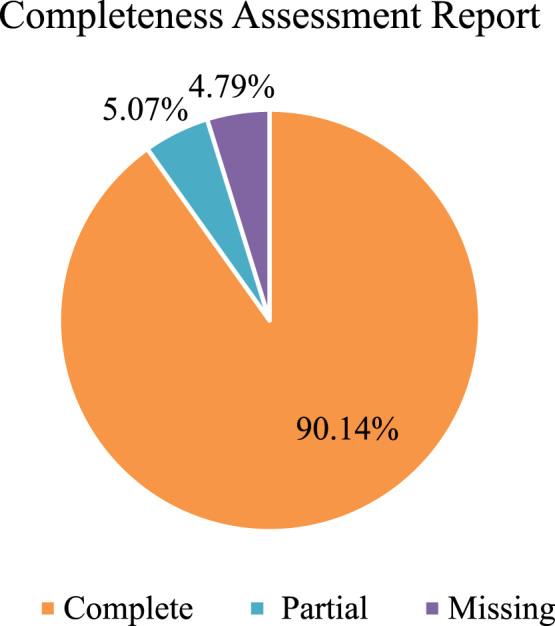
Representation of completeness scores of *Aegle marmelos* leaf transcriptome assembly.

The quality of raw reads was checked for the ambiguous bases, phred Score (>30), read length, nucleotide base content and other parameters via FASTQC. A total of 115.92 million good quality reads were obtained after the removal of low-quality reads. The transcripts of length 200 bp and above were retained for further analysis. The De novo assembly of high-quality reads was performed using Trinity (version: 2.3.2) with default parameters and K-mer size of 25. *Aegle marmelos* leaf sample produced a total of 133,616 trinity transcripts clustered into 46,345 unigenes and with GC percentage of 40.50 (Table 1). Further to predict the completeness of transcriptome assembly "Bench-marking universal single-copy orthologs” (BUSCO) software (v3) was used in the gVolante server [8]. The analysis showed that out of the total 1440 core gene queried for plants 1371 (95.21%) were completely or partially present (Fig. 1). While the average number of ortholog per gene was 2.03 and there were 64.33 percent of genes with more than one ortholog (Table 2).

**Table 1:** Assembly statistics of *Aegle marmelos* leaf.

Parameters	Statistics
Total trinity unigenes	46,345
Total trinity transcripts	133,616
Percent GC	40.50
Contig N10	5085
Contig N20	4063
Contig N30	3412
Contig N40	2925
Contig N50	2544
Median contig length	1395
Average contig	1691.19
Total assembled bases	225,969,847

**Table 2:** Completeness assessment report.

Parameters	Statistics
Total number of core genes queried	1440
*Number of core genes detected*	
Complete	1298 (90.14%)
Complete + Partial	1371 (95.21%)
Number of missing core genes	69 (4.79%)
Average number of orthologs per core genes	2.03
% of detected core genes that have more than 1 ortholog	64.33
