# Supplementary material for: Data of de novo assembly of the leaf transcriptome in Aegle marmelos
Source: Data Brief. 2018 May 23;19:700–3. doi: 10.1016/j.dib.2018.05.095 (PMC6139533; doi:10.1016/j.dib.2018.05.095)
Supplement: Supplementary file 1 — Supplementary material [file mmc1.doc]

Conflict of Interest and Authorship Conformation Form

Please check the following as appropriate:

- All authors have participated in (a) conception and design, or analysis and interpretation of the data; (b) drafting the article or revising it critically for important intellectual content; and (c) approval of the final version.
- This manuscript has not been submitted to, nor is under review at, another journal or other publishing venue.
- The authors have no affiliation with any organization with a direct or indirect financial interest in the subject matter discussed in the manuscript

Author’s name Affiliation

Prashant Kaushik Instituto de Conservación y Mejora de la Agrodiversidad Valenciana, Universitat Politècnica de València, Valencia, Spain
